# Diseases of the Kidney

**Published:** 1901-11-16

**Authors:** 


### DISEASES OF THE KIDNEY.

(*Continued from page* 101.)

**Urology.**—T. Calvert [15] reports a case of hæmatoporphyrinuria in a woman aged 32. There had been dyspepsia and hæmatemesis, and the urine contained no albumen, sugar, or blood, but was dark from the porphyrin present. A previous attack occurred a year before, but no cause could be discovered. Another instance of hæmatoporphyrinuria was found recently by Nicolaysen [16] after an operation on appendicitis under chloroform. The condition had not been present before and quickly passed away, leaving the urine normal except for some urobilin. Another rare condition, renal colic, due to the passage of casts of the ureters is mentioned by J. A. H. White.[17] After one of many attacks of colic the urine contained blood and mucoid casts about an inch long. Rapid improvement took place when iodide of potash was given to render the secretion more fluid.

**Albumosuria.**—Hamburger [18] had a patient suffering from multiple sarcomata of the bones. The urine was copious, pale, and gave a strong biuret reaction. A few drops of nitric acid gave a precipitate which disappeared on boiling and returned on cooling. On heating the urine slightly a precipitate was also found which vanished on adding acetic acid, or boiling, but reappeared on cooling. Hougounenq [19] contrasts with this a case where no cloud was produced by boiling or heating gently, though the other signs of albumose were present. The former type of reaction is rare and indicative of bone lesions, while the latter is found in nephritis, pregnancy, insanity, and other states. Kalischer [20] met with another instance of this "Bence Jones" reaction in connection with myeloma of the ribs. There was some tenderness and pain over the ribs at certain spots, and dyspnœa and general weakness. The urine gave a dense cloud on heating to 60° which vanished on boiling and reappeared on cooling. If nitric acid was added to a cold specimen the ring appeared, which also vanished during boiling. Occasionally true albumen also appeared. After death the ribs were found widely affected by myeloma. T. R. Bradshaw [21] notices that in a case observed from a very early stage the albumose was at first present in minute quantities, but gradually increased to 1 per cent. It precedes all other signs of the disease and is a most important sign for the diagnosis of this form of malignant growth. Askanazy,[22] however, found "Bence Jones" albumose in the urine of a patient who died from lymphatic leukæmia. The bone marrow contained albumose. Yet there were no bone tumours, but a number of glandular ones. It is clear that either the albumose was merely the form which does not precipitate on heating to which Hougounenq refers, or else the rare form is sometimes the result of lymphoid change in the marrow as well as of myelomata. *Ureine.*—The discovery of an organic liquid body, the cause of the principal reactions of urine, has been strongly criticised by Chace and Gies,[23] who insist that it is merely a mixture of many of the ordinary constituents of urine, and that Dr. Moor's chemical methods of separating it are unreliable. *Bacteriuria* is due either to organisms such as pus cocci and the proteus which decompose urea and cause alkaline urine, or to the group comprising tubercle, typhoid, colon bacilli, and the gonococcus which do not break up urea, and leave the urine acid. The most common cause of cystitis is the B. coli, and the infection, whatever the organism is, may travel down the urinary tract from the blood or ascend from the urethra.[24] Two interesting cases of bacteriuria are discussed by A. W. Williams and R. J. Wilson.[25] The first patient was an old woman with periodic attacks of mental failure followed by the secretion of quantities of cloudy urine containing the B. aërogenes. The patient was rapidly sinking when surgical interference was advised, and the kidney opened and drained, with the result that complete recovery took place. Here there was periodic occlusion of a ureter causing retention of bacterial toxins which were removed by free drainage. In the second patient the necessity of an exact diagnosis was clearly shown. The urine contained pus and B. coli, and as there was lumbar pain and some pyrexia it was supposed that pyelitis was present. However, catheterisation of the ureters showed that the infection was confined to the bladder, operation was negatived, and after lavage of the bladder the patient recovered. *The bacillus coli* exists under so many different conditions that its real importance has been a matter of much discussion. One of the latest investigations is that carried out with much skill by Kenneth M. Douglas [26] on its relation to cystitis. His conclusions are that it is present in the bulk of cases, and in many it seems to be the cause of the disease. Still it is found sometimes in the bladder for long periods without bringing about cystitis. In some cases where this bacillus is found, it seems to be the supplanter of other organisms rather than the initiator of the disease. Its extraordinary variations in form and virulence have led to much confusion, and no one culture reaction is sufficient to enable us to identify it with certainty. *Cyclic albuminuria.*—The editor of the "Medical Record," in discussing the views of Sutherland and Hawkins as to its causation,[27]

makes the important criticism that if postural albuminuria is due to variation of blood pressure, how is it that the theory is not tested by actual measurement of the pulse tension at various hours? At present no one knows with certainty whether it is functional or physiological or tending to an organic change in the kidney. *Cells in the urine.*—G. Leslie Eastes[28] finds that the rodded granular epithelial cells of the convoluted tubules with extended angular processes are present chiefly in acute or sub-acute nephritis which is passing into a late stage. In carcinoma he has noticed renal cells in large clumps dovetailed together, as well as blood and cells of the renal pelvis. These latter cells appear also in cases of calculi and pyelitis. They are granular, have a large nucleus and prolongations at one or both ends two or three times the length of the cell. Calculi produce also hyaline casts and a few blood cells and crystals; pyelitis shows pus and broad casts as well as cells of the pelvis. The cells from the bladder are much larger than those from elsewhere; in cystitis they are chiefly squamous; in epithelioma the cells are smaller and come away in groups; in papilloma we have very long thin acicular cells. W. T. Howard[29] remarks that in acute interstitial nephritis there are present Unna's plasma cells, lymphocytes, and eosinophiles, with one or several nuclei. Tarchetti[30] inquires why red corpuscles are so often decoloured in urine. This is most noticeable in urine which is dilute or deficient in chlorides, but even in normal urine there is usually some loss of colour; yet this is not due to length of sojourn in the urine. The fact probably is that the urine as secreted by the glomeruli is extremely dilute, and has little or no chloride, but becomes concentrated in the tubules. Hence the red cells are decolourised before they reach the bladder from the comparative absence of salt in its earliest stage.

[15] Treatment, March. [16] Brit. Med. Jour., Feb. 16. [17] Treatment, March. [18] Med. Rec., April, and Johns Hopkins Bullet., Feb. [19] Brit. Med. Jour., Feb. [20] Amer. Jour. of Med. Sciences, June. [21] Brit. Med. Jour., July 13. [22] Birmingh. Med. Rev., April. [23] Med. Rec., March 2. [24] Clinical Jour., July 24. [25] Med. Rec., Feb. [26] Scottish Med. Jour., Feb. [27] Med. Rec., Ap. 20. [28] Brit. Med. Jour., March 9. [29] Brit. Med. Jour., March 30. [30] Brit. Med. Jour., Oct. 30.

